# Predicting El Niño Beyond 1-year Lead: Effect of the Western Hemisphere Warm Pool

**DOI:** 10.1038/s41598-018-33191-7

**Published:** 2018-10-08

**Authors:** Jae-Heung Park, Jong-Seong Kug, Tim Li, Swadhin K. Behera

**Affiliations:** 1 International Pacific Research Center and Department of Atmospheric Sciences, School of Ocean and Earth Science and Technology, University of Hawaii at Manoa, Honolulu, Hawaii USA; 2 Division of Environmental Science and Engineering, Pohang University of Science and Technology (POSTECH), 37673 Pohang, Korea; 3 Key Laboratory of Meteorological Disaster, Ministry of Education (KLME)/Joint International Research Laboratory of Climate and Environmental Change (ILCEC)/Collaborative Innovation Center on Forecast and Evaluation of Meteorological Disasters (CIC-FEMD), Nanjing University of Information Science and Technology, Nanjing, China; 4 Application Laboratory, Japan Agency for Marine-Earth Science and Technology, Yokohama, Kanagawa Japan

## Abstract

Due to the profound impact of El Niño-Southern Oscillation (ENSO) on global climate and weather, extensive research has been devoted to its prediction. However, prediction accuracy based on observation is still insufficient and largely limited to less than one year of lead time. In this study, we demonstrate the possibility that anomalous sea surface temperature (SST) warming (cooling) in the Western Hemisphere Warm Pool (WHWP, a.k.a. Atlantic Warm Pool) near the Intra-Americas Sea (IAS), which is the second largest warm pool on the planet, contributes to the initiation of La Niña (El Niño) with a 17-month lag time. SST anomalies in WHWP in late boreal summer contribute significantly to the emergence of the Pacific meridional mode (PMM) via interaction between the ocean and atmosphere over the subtropical North Pacific during the subsequent winter and spring. Near-equatorial surface wind anomalies associated with the PMM can further trigger ENSO through the dynamics of the equatorial oceanic waves. Thus, this observational analysis presents a clear step-by-step explanation about the influence of WHWP on ENSO development with a 17-month lead time.

## Introduction

The El Niño-Southern Oscillation (ENSO) exerts considerable influence on weather and climate around the world via atmospheric teleconnection^1–3^ even though it occurs in the equatorial Pacific. Hence, extensive studies have been conducted based on observational data to better understand the triggering mechanisms of El Niño^4–6^ (a positive phase of ENSO) and associated precursors^7,8^ that help in anticipating the El Niño occurrence and its accompanied extreme climate events. For instance, some previous studies emphasized the role of warm water volume (WWV)^9^ in the equatorial Pacific as an essential precondition of ENSO. Since the ocean’s memory is dependent on the wave adjustment process according to the delayed oscillator theory^10^ or the recharge oscillatory theory^11,12^, a WWV peak usually precedes the El Niño by approximately eight months^13^. In addition, precursory signals from extratropical Pacific regions, such as the North Pacific Oscillation during boreal winter (for convenience, seasons in this study follow those in the Northern Hemisphere), can also lead to subsequent El Niño development via a seasonal foot-printing mechanism^14^ (SFM). Similarly, the Pacific meridional mode (PMM)^15,16^ in the subtropical North Pacific and its related trade winds can induce a charging mechanism^17,18^, which is known as another precursor that will often trigger El Niño events.

Atmospheric teleconnection from other oceans may also help to trigger El Niño. For example, Atlantic Niño, which peaks in summer, may independently enhance El Niño development by modulating the Walker Circulation in the Atlantic and eastern Pacific sector^19–21^. Ham *et al*.^20^ proposed that a negative sea surface temperature (SST) anomaly in the tropical North Atlantic (TNA) during early spring could induce El Niño development via Rossby wave response to an anomalous heat source in the TNA and local air–sea interactions along the intertropical convective zone (ITCZ) in the subtropical North Pacific, which could be used to predict El Niño with a three-season lead time. Regarding the influence of the TNA, Yu *et al*.^21^ mentioned that a cold SST anomaly (SSTA) in the TNA in summer could force a remote westerly anomaly response in the equatorial Pacific through atmospheric Kelvin wave response and the Indian Ocean relaying effect.

Izumo *et al*.^22^ argued that the Indian Ocean dipole (IOD)^23^, which has a peak phase in fall, may trigger El Niño by modulating surface wind anomalies over the western equatorial Pacific in the following spring and summer. It was emphasized that the effect of IOD could help predict El Niño events with a 13 to 15-month lead time. These results imply that interbasin interaction can play a key role in El Niño prediction beyond one year. Such a long-lead relation is of great importance as it overcomes the spring predictability barrier of El Niño^24^ which makes El Niño predictions beyond one year very difficult.

In addition, some forecast experiments have shown that the interbasin interactions with the Indian and Atlantic Oceans can improve ENSO prediction^25^. These observational and modeling studies motivated us to consider other possible precursors in the Atlantic Ocean that could extend El Niño prediction lead time. In this light, we suggest that an SSTA in the Western Hemisphere Warm Pool^26^ (WHWP, a.k.a. Atlantic Warm Pool), which is located in the Intra-Americas Sea (IAS), is a new independent precursor of El Niño with a forecast lead time of 17 months.

## Results

### Precursory components of El Niño with a 17-month lead

**Figure 1:**
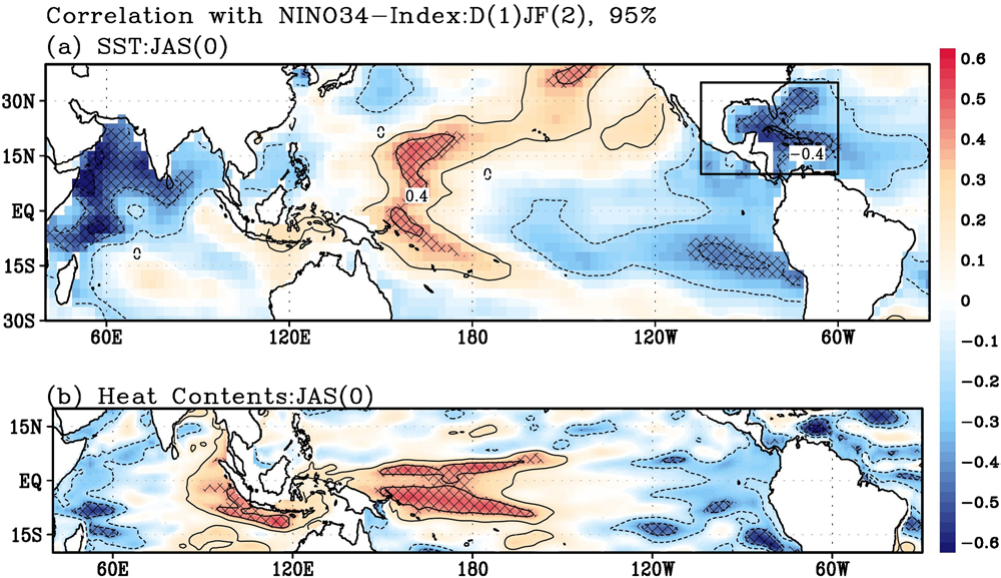
Relation between SST/HC and Nino3.4 index. Lead-lag correlation maps of (**a**) SST and (**b**) HC anomalies in JAS(0) based on the Niño3.4 index in D(1)JF(2), indicating 17-month lead-lag, are shown. The WHWP region is indicated by the black box in (**a**). Contour intervals are 0.2, and hatching indicates 95% confidence level by two-tailed t-test.

A lead-lag correlation analysis with Niño3.4 index from December (1) to February (2) (i.e., D(1)JF(2), where the number denotes the sequential order of year) revealed that there is a significant positive correlation between SST and heat content (HC) anomalies over the tropical western Pacific ~17 months prior (July to September, JAS(0)) to an El Niño peak in winter (Fig. 1). Note that HC is closely related to the WWV. Herein, HC is defined as the vertical average of oceanic temperature in the upper 300 m^27^ (refer to Methods). Figure 1 indicates that the configuration of SSTA in the western Pacific is an ocean surface manifestation of HC in the western equatorial Pacific. Thus, the HC in the western equatorial Pacific should be considered as a long-lead representative precursor of ENSO, as indicated in a previous study^28^.

In addition to the Pacific signal, a significant SST cooling signal appears in the western Indian Ocean, a region often used to represent the western pole of the IOD^23^. Together with weak SST warming near the Sumatra in the eastern Indian Ocean, the SST pattern in the Indian Ocean can be considered as an IOD in JAS(0), which is in the middle of developing  process into a peak phase of the IOD in fall^29^. According to the previous studies^29–31^, the IOD evolves into a basin-wide cooling pattern during the following winter. The basin-wide cooling in the Indian Ocean persists from winter to the following summer, and it induces anomalous westerly winds in the western equatorial Pacific through Kelvin wave response^31,32^ and establishes a low-level cyclonic circulation anomaly. Since the westerly wind anomalies over the western equatorial Pacific is known to provide favorable conditions for El Niño development, an IOD may be regarded as a potential long-lead precursor of El Niño. However, it should be emphasized that during JAS(0) an SSTA over the western Indian Ocean, rather than the eastern Indian Ocean, plays a more significant role in subsequent El Niño development or phase transition^30^, as inferred in Fig. 1a.

**Figure 2:**
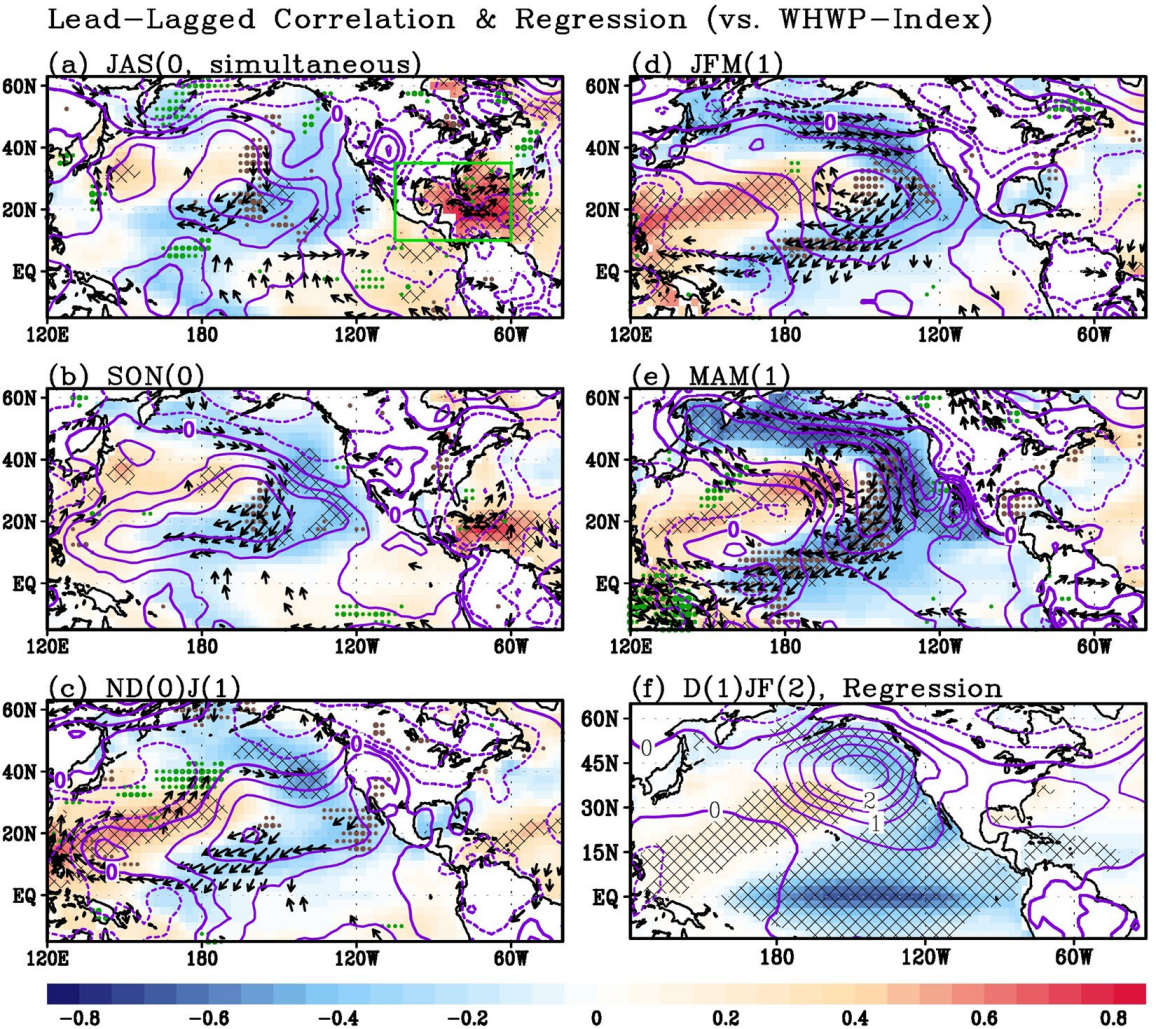
Lagged influence of WHWP–SST on the Pacific. Lagged correlation maps of SST (shading; above 90% confidence level is hatched), SLP (contour; interval is 0.2), wind (vectors; shown only above 90% confidence level) and precipitation (dots; positive-green, negative-brown; shown only above 90% confidence level) anomalies in (**a**) JAS(0), (**b**) SON(0), (**c**) ND(0)J(1), (**d**) JFM(1), and (**e**) MAM(1) based on the WHWP (JAS0) index. For (**f**), regression results against normalized the WHWP index in D(1)JF(2) are shown, where contour interval of SLP anomaly is 0.5hPa.

Interestingly, a significant negative SSTA signal is found in the IAS and far eastern Pacific. These regions are known as WHWP^26^, which is the second largest warm pool on the planet. The statistical significance of this long-lead (about six seasons) correlation between WHWP and El Niño suggests that interbasin interaction exists between the Pacific and Atlantic Oceans, as well as the Pacific and Indian Oceans. It is necessary to consider significant SST signals over the subtropical eastern South Pacific and tropical Atlantic. However, since the signals are small and possibly related to WHWP through atmospheric circulation, we focus on WHWP. Therefore, WHWP–El Niño connection provides an additional precursory signal for possible prediction of El Niño with a lead time greater than one year. It should be noted that the long-lead relation between WHWP and El Niño has become significant during the past three decades (Supplementary Fig. 1). Such an interdecadal change could be attributed to the recent increase in climatological SST in the tropical Atlantic^33,34^. To validate this information, we conducted modeling experiments using GFDL-CM2.1, where recent climatological SST warming in WHWP in JAS season is forced (see Supplementary Fig. 2 for more information). In the experiments, a higher climatological SST in WHWP in JAS season resulted in more significant connection between WHWP and Pacific; this result is consistent with the results presented in Fig. 2. These results support the idea that the recent climatological warming in WHWP plays an important role in the WHWP–El Nino connection.

### Influence of WHWP on the Pacific

The abovementioned observational results point out three key precursory signals for El Niño with ~17-month lead time. The first is the HC over the western equatorial Pacific. The second and the third are IOD and WHWP, respectively. Since the relation between precursory HC and IOD signals and El Niño have been previously reported^22,28^, this study focuses on the mechanism through which SSTA in WHWP affects El Niño development. To examine specific processes through which WHWP influences an SSTA in the equatorial Pacific, lagged correlation/regression analysis is conducted in various seasons based on the WHWP index (Fig. 2) (see Method in terms of index, Supplementary Fig. 3). As illustrated in Fig. 2a, a strong surface warming in WHWP during JAS(0) induces a simultaneous negative sea level pressure (SLP) anomaly *in situ* and a positive SLP anomaly (SLPA) over the subtropical northeastern Pacific^35^. Note that SST amplitude and areal expansion of WHWP are climatologically greatest during JAS season. Thus, influence of WHWP on the atmospheric circulation is expected to be greatest during this season. If one considers JJA (June to August) or ASO (August to October) instead of JAS, a similar result can be obtained.

A SST warming in the WHWP leads to a low-level anomalous cyclone to the west of the anomalous heat source in the WHWP due to Rossby wave response^36^ (Supplementary Fig. 4). The northerly wind anomalies to the west of the low-level cyclone advect negative moist static energy (MSE) into the subtropical North Pacific, which leads to negative precipitation anomalies over the region. The northerly wind anomalies also enhance the mean northeasterly trade winds in the region, leading to an increase in latent heat flux and thus SST cooling (Fig. 2a). Meanwhile, the SST cooling and the suppressed precipitation over the subtropical North Pacific induce an anomalous anticyclone to the west according to the off-equatorial Gill response and atmosphere–ocean interaction in the subtropics^37^. The enhanced northerly wind anomalies associated with the anomalous anticyclone further strengthen the negative MSE advection and associated precipitation anomalies. Through this positive feedback, the local cold SSTA and the positive SLPA are maintained throughout the subsequent winter and spring even after the dissipation of SSTA in WHWP (Fig. 2b–e).

While the climatological trade winds persist over the central and eastern North Pacific throughout the year, which is critical to maintaining the local cold SSTA (Fig. 2a–e), the seasonal change of the background winds from JAS(0) to JFM(1) (January to March) near the coast of the East Asia is responsible for the formation of a northeast-southwest tilted warm SSTA belt in the northwestern Pacific during winter. Since climatologically northerly mean winds prevail near the coast of the East Asia in this season, an anomalous subtropical anticyclone over the subtropical North Pacific induces anomalous SST warming over its western region with less latent heat fluxes from the ocean’s surface due to a reduction in total wind speed. In addition, the anticyclonic flows deepen the thermocline due to anomalous Ekman downwelling, which results in SST warming^38^. The anomalous subtropical anticyclone is closely coupled with the dipole SSTA pattern, with a tilted warm (cold) SSTA belt to the west (east). The subtropical dipole SSTA pattern becomes strongest in MAM(1) (March to May), resembling a typical PMM pattern (Fig. 2e).

The full development of PMM is accompanied by the southwestward propagation of SSTA from the subtropics toward the Tropics due to wind–evaporation–SST feedback^15^. During the southward propagation, seasonal ITCZ provides an environment for active air–sea interaction. Hence, a small perturbation initiated in the subtropics can be intensified when the ITCZ is present. As the subtropical signals of the PMM reach to the equatorial region, the easterly anomalies force oceanic upwelling of Kelvin waves and trade-wind discharging^17,18^, leading to the development of La Niña in the subsequent seasons (Fig. 2f).

The observational analysis above reveals the significant long-lead impact of WHWP on SSTA in the equatorial Pacific. Model simulation results are also used for the period from 1970 to 2000 to evaluate the performance of climate models participated in the Coupled Model Inter-comparison Project phase 5 (CMIP5) (Supplementary Table 1). Consistent with the observational analysis, we first checked whether SST in WHWP is capable of influencing subsequent El Niño in each model simulation. It is found that in terms of the correlation between SST in WHWP and Niño3.4 index (Supplementary Fig. 5), most of the CMIP5 models (87.8%; 29/33) reproduced the negative WHWP–ENSO correlation, which is consistent with the observation. In particular, 15 models show significant correlation at a 95% confidence level (Supplementary Table 1). Further analysis reveals that the models tend to simulate the evolution of the teleconnection patterns from WHWP to ENSO reasonably for approximately six seasons (Supplementary Fig. 6). In particular, with respect to the abovementioned 15 models, anomalous northerly winds and SST cooling over the North Pacific during summer to winter were identified (Supplementary Fig. 7). In turn, PMM-like and La Niña patterns are shown in MAM(1) and OND(1) (October to December) seasons, respectively. This result warrants a further examination of the CMIP5 models.

### El Niño Prediction with a multiple regression model

Thus far, we have discussed the possible role of WHWP in El Niño generation with a lag time greater than one year. Given the physical linkage between WHWP and El Niño, SSTA in the WHWP can be used as a precursor for long-lead El Nino prediction. To validate this possibility further, we applied the Granger causality analysis^39,40^. From this analysis, SSTA in the WHWP is proved to be independent from previous El Niño and able to trigger El Niño with 17-month lag at a 95% confidence level (Supplementary Table 2). A simple statistical model is constructed using the preceding WHWP signal and other precursory signals, such as HC in the equatorial western Pacific and IOD, as shown in Fig. 1. The IOD and WHWP indices change slightly based on whether the previous El Niño signals are retained or removed; thus, the result in this study is not sensitive to the effects of previous El Niño, even though El Niño signals are removed from both indices.

**Figure 3:**
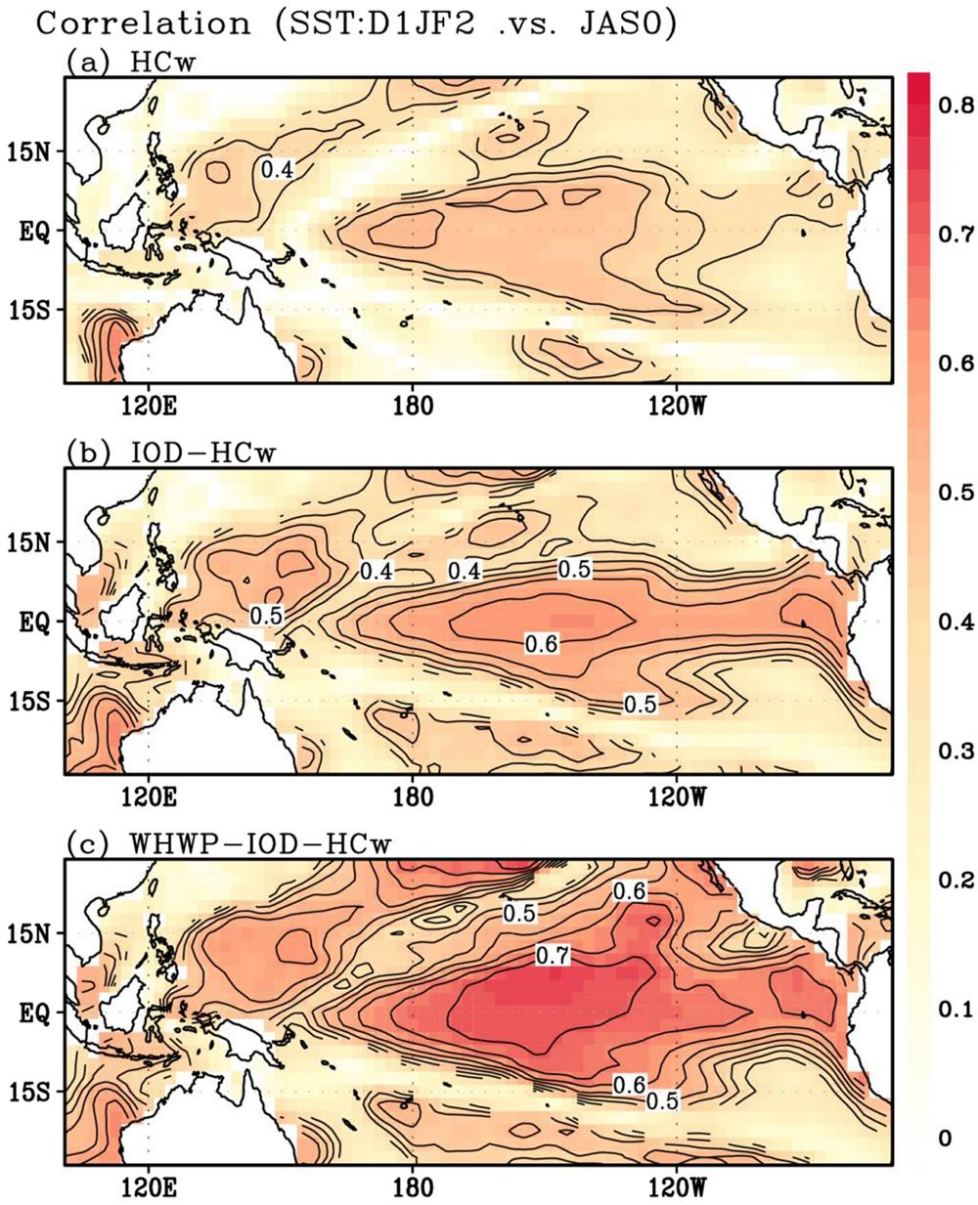
Grid-to-grid correlation maps between observed SSTA and reproduced SSTA (Observation). For SST reconstruction, (**a**) HC index only, (**b**) IOD and HC indices, and (**c**) WHWP, IOD, and HC indices are used in multiple regression method, respectively. Contour intervals are 0.05.

We first examined the influence of equatorial HC, which considers internal dynamics in the Pacific. By regressing the HC index (JAS(0)) onto the observed SSTA (D(1)JF(2)), SSTA fields are reconstructed. Then, the correlation is calculated at each grid point between the observed and reconstructed SSTA, as shown in Fig. 3a (similar results for another season can be seen in Supplementary Fig. 8). The highest correlation (~0.5) appears over the equatorial Pacific. In a linear sense, this implies that ~25% of the total SSTA variability can be explained by the Pacific HC. Next, in addition to the Pacific HC, the preceding IOD effect is considered via multiple regression analysis. The correlation coefficients between the reconstructed SSTA field and the observed SSTA field are shown in Fig. 3b. As depicted in the figure, the maximum correlation coefficient increases to ~0.62, which indicates that up to 38% of the total variance can be explained from a linear perspective. Compared to Fig. 3a, the correlation pattern in Fig. 3b appears more symmetrical about the equator, which is indicative of the effect of previously discussed equatorially trapped waves generated by IOD.

Lastly, the effect of WHWP is incorporated into the multiple regression model (Fig. 3c) and then a marked increase in the correlation is obtained. The peak correlation coefficient increases to 0.75, which appears slightly off the equator, reflecting PMM influence. Such a high correlation coefficient means that up to 56% of the ENSO variability can be captured by the combined WHWP–HC–IOD precursory signals. This variance is much higher than that from the combined HC–IOD effect (38%), possibly due to the independence of WHWP. A cross validation (excluding one of their time series) further revealed that correlation coefficients against the observed SSTA exceed 0.60 in the Niño3.4 region, exceeding a 99% confidence level (Supplementary Fig. 9). Herein, multiple regression coefficients of the WHWP, IOD, and HC indexes vs. the Niño3.4 index are 0.43, 0.39, and 0.31, respectively, indicating that 42.7%, 35.1%, and 22.2% of the reproduced variability can be explained by those three precursory components.

It is worth noting that the increase in the correlation coefficient appears not only in the equatorial Pacific but also in the subtropical western North Pacific. This is understandable because the two regions are dynamically linked via westward propagating Rossby waves^41^. Because the western North Pacific is recognized as a key region that conveys El Niño impacts, the WHWP effect appears to provide an additional benefit in the prediction of El Niño.

**Figure 4:**
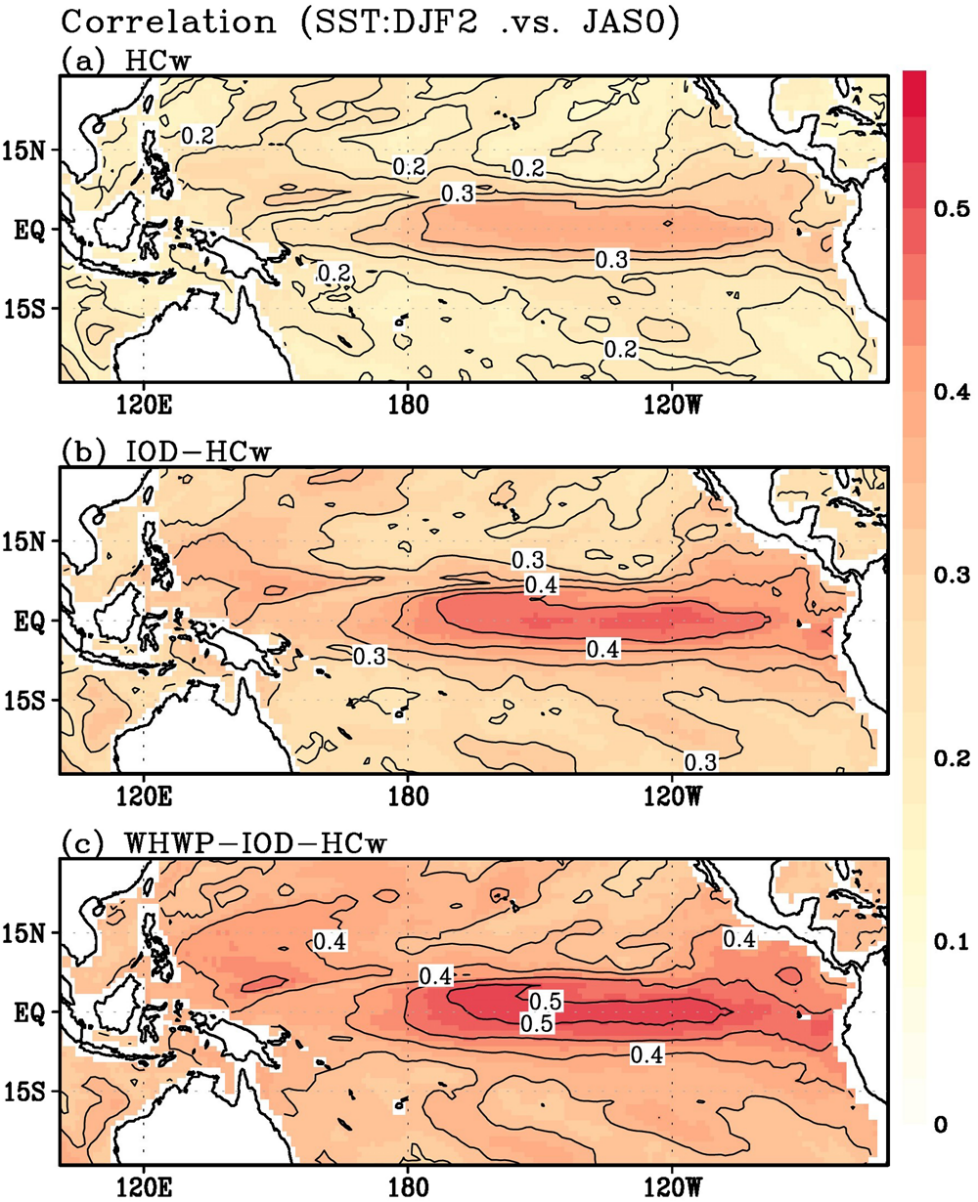
Grid-to-Grid correlation maps between the observed SSTA and reproduced SSTA (CMIP5). The same figures to Fig. 4, but ensemble result from historical simulation runs (1970–2000) in CMIP5 models.

Based on the significant relation between WHWP and ENSO, we examined how WHWP is related to the ENSO event, combined with the IOD and HC in the CMIP5 long-term simulations. For this, the same calculation applied to the observation is repeated. Figure 4 shows the ensemble average of correlation of 28 models that correctly captures the lagged WHWP–ENSO relation. As expected, the combination of WHWP with the HC and IOD precursory signals leads to a noticeable increase in the correlation coefficient. Analytical results from individual models also show the possibility that WHWP can improve the El Niño prediction beyond 1-year of lead time (Supplementary Fig. 10).

## Discussion

By analyzing observational and model simulation data, we showed a significant influence of WHWP on El Niño with a 17-month lead time. Results of our evaluation indicated an increase of an SSTA in the WHWP in mid-summer to early-fall induces a northerly wind anomaly over the North Pacific as a result of Rossby wave propagation, which in turn generates a cold SSTA and a high SLPA in the subtropical northeastern Pacific. The cold SSTA and high SLPA together with associated anomalous low-level anticyclone persist through the subsequent winter and spring due to positive air–sea feedback. The equatorward expansion of the cold SSTA and the anomalous circulation in the subtropical North Pacific lead to the generation of a PMM-like pattern in the spring, triggering a La Niña through induced equatorial easterlies that force upwelling Kelvin waves and trade-wind discharging of equatorial heat content^17,18^. Thus, the occurrence of a PMM-like pattern in the northeastern subtropical Pacific plays a key role in linking WHWP to ENSO.

This robust relation among WHWP, PMM, and ENSO can provide a longer lead time, compared to a lead time reported in previous studies (such as Izumo *et al*.^22^ that emphasized the effect of the IOD). A multiple regression model was constructed to demonstrate the significant value added to El Niño prediction by including WHWP influence. It has been shown that the combination of the WHWP signal with equatorial HC and IOD signals leads to much better predictions throughout the entire tropical Pacific basin (Figs. 3 and 4). This physics-based empirical model can thus extend the limits of current dynamical models and overcome the spring predictability barrier.

The WHWP–ENSO relation experienced a marked interdecadal change (Supplementary Fig. 1); actually, the relation was not statistically significant before 1985, which suggests a weaker Atlantic influence on the Pacific in the previous period. The relation has become more robust in the last three decades. Regarding this, we found that climatology of SST in the WHWP is greater than 28.5 °C in recent decades, keeping paced with the SST warming trend in the Atlantic, global warming, or both, which appears to be responsible for this inter-decadal change through deep convection and related atmospheric teleconnection. These aspects will be covered in our future research.

Unfortunately, some of CMIP5 models underestimate the lagged relation between WHWP and El Niño (Supplementary Fig. 5), which is consistent with the fact that climate models tend to underestimate the Atlantic influence on the tropical Pacific^42,43^. This is caused by either mean SST biases over the northeastern Pacific near the western coast of Mexico where the PMM develops or the error in the mean circulation over WHWP. Hence, the relation between the regional mean state error and the interannual variability must be examined in detail. If a model is able to capture the WHWP–ENSO teleconnection pattern, the model predictability of El Niño and associated extreme events could be extended beyond one year.

## Method

### Observational data

In this study, several reanalysis datasets were used to analyze the relation between WHWP and El Niño. The analysis covers the period from 1985 to 2016. The SST data used in this analysis were the Extended Reconstructed Sea Surface Temperature version 3 (ERSSTv3)^44^ from NOAA, which was constructed based on a statistical interpolation of the International Comprehensive Ocean–Atmosphere Data Set (ICOADS) version 2.4. Atmospheric data, including wind and SLP fields, were obtained from the NCEP/NCAR Reanalysis 1 (NCEP-R1)^45^. The precipitation data were from the Global Precipitation Climatology Project version 2.1 (GPCPv2.1)^46^, which has a horizontal resolution of 2.5° × 2.5°. For subsurface oceanic data, Global Ocean Data Assimilation System is used^47^ (ftp://ftp.cdc.noaa.gov/Datasets/godas). Using this data, HCs were calculated.

### Index

HC is defined as a vertical average of oceanic temperature within upper 300 m^27^. We defined the HC index as an area average of the HC anomaly in the western Pacific (140°E-150°W, 6°S-6°N) during the JAS season. For the IOD index, the areas for the IOD are 50°–70°E within 10°S–10°N for west and 90°–110°E within 10°S-0° for east in JAS season, and the IOD index is defined as an areal-averaged SST difference (west minus east). The WHWP index is obtained by areal average of SSTA in 60°–105°W within 10°–35°N during the JAS season. With respect to WHWP and IOD indices, previous El Niño signal (Niño3.4 in DJF season) is removed by partial regression since previous ENSO is able to affect the subsequent SSTA in the Atlantic and the Indian Oceans.

### CMIP5

The outputs of 33 CMIP5 models from historical (1970–2000) experiments (with one ensemble member each) were used (Supplementary Table 1.) For convenience, a sea surface height (SSH) field was used as a proxy to represent the HC variation. For the SSH, only 28 model outputs were used due to data limitations of some models. Before the lagged regression analysis, both the linear trend and the climatological annual cycle were removed and a three-month running average was applied to reduce the effect of high-frequency variations and to focus on the interannual variability.

## Electronic supplementary material

Supplementary information
