# Supplementary material for: Predicting El Niño Beyond 1-year Lead: Effect of the Western Hemisphere Warm Pool
Source: Sci Rep. 2018 Oct 8;8:14957. doi: 10.1038/s41598-018-33191-7 (PMC6175942; doi:10.1038/s41598-018-33191-7)
Supplement: Supplementary file 1 — Supplementary information [file 41598_2018_33191_MOESM1_ESM.docx]

**<Supplementary Information>**

**Predicting El Niño Beyond 1-year Lead:
Effect of Western Hemisphere Warm Pool**

Jae-Heung Park^1^, Jong-Seong Kug^2^, Tim Li^1,3^, and Swadhin K. Behera^4^

^1^International Pacific Research Center and Department of Atmospheric Sciences, School of Ocean and Earth Science and Technology, University of Hawaii, Honolulu, Hawaii 96822

^2^Division of Environmental Science and Engineering, Pohang University of Science and Technology (POSTECH), 37673 Pohang, Korea.

^3^Nanjing University of Information Science and Technology, Nanjing, China

^4^Application Laboratory, Japan Agency for Marine-Earth Science and Technology, Yokohama, Kanagawa, Japan

**Supplementary Figure & Table**

**Supplementary Table 1.** 33-models used in this study from the historical experiments in CMIP5. Model names in bold indicate the models that show good relationship between WHWP and ENSO, consistent to observational analysis based on the Supplementary Fig. 5.

| **Model Name** | **Modeling Center (or Group)** |
| --- | --- |
| ACCESS1-0 | CSIRO (Commonwealth Scientific and Industrial Research Organisation, Australia), and BOM (Bureau of Meteorology, Australia) |
| BCC-CSM1-1 | Beijing Climate Center, China Meteorological Administration |
| BCC-CSM1-1-m |  |
| **CanESM2** | Canadian Centre for Climate Modelling and Analysis |
| **CCSM4** | National Center for Atmospheric Research |
| **CESM1-CAM5** | National Science Foundation, Department of Energy, National Center for Atmospheric Research |
| CNRM-CM5 | Centre National de Recherches Meteorologiques |
| **CMCC-CESM** | Centro Euro-Mediterraneo per I Cambiamenti Climatici |
| CMCC-CM |  |
| **CMCC-CMS** |  |
| **CSIRO-Mk3-6-0** | Commonwealth Scientific and Industrial Research Organisation in collaboration with the Queensland Climate Change Centre of Excellence |
| EC-EARTH | EC–EARTH consortium |
| FGOALS-g2 | LASG, Institute of Atmospheric Physics, Chinese Academy of Sciences; and CESS, Tsinghua University |
| FGOALS-s2 | LASG, Institute of Atmospheric Physics, Chinese Academy of Sciences |
| **FIO-ESM** | The First Institute of Oceanography, SOA, China |
| GFDL-CM3 | NOAA Geophysical Fluid Dynamics Laboratory |
| GFDL-ESM2G |  |
| GFDL-ESM2M |  |
| GISS-E2-H | NASA Goddard Institute for Space Studies |
| GISS-E2-R |  |
| HadCM3 | Met Office Hadley Centre (additional HadGEM2-ES realizations contributed by Instituto Nacional de Pesquisas Espaciais) |
| HadGEM2-AO | Met Office Hadley Centre |
| **HadGEM2-CC** |  |
| HadGEM2-ES |  |
| **INMCM4** | Institute of Numerical Mathematics, Russian Academy of Sciences |
| IPSL-CM5A-LR | Institute Pierre Simon Laplace |
| **MIROC5** | Atmosphere and Ocean Research Institute (The University of Tokyo), National Institute for Environmental Studies, and Japan Agency for Marine–Earth Science and Technology |
| **MIROC-ESM** | Japan Agency for Marine–Earth Science and Technology, Atmosphere and Ocean Research Institute (The University of Tokyo), and National Institute for Environmental Studies |
| MPI-ESM-LR | Max Planck Institute for Meteorology |
| **MRI-CGCM3** | Meteorological Research Institute |
| **MRI-ESM1** |  |
| **NorESM1-M** | Norwegian Climate Centre |
| **NorESM1-ME** |  |

**Supplementary Table 2.** Multiple-regression analysis associated with Granger causality. Multiple-regression coefficients of Niño3.4 and WHWP indices in both D(-1)JF(0) and JAS(0) seasons against Niño3.4 index in D(1)JF(2). In (a), both Niño3.4 and WHWP indices are used at the same time, and in (b) Niño3.4 index is adapted only. While the explained variance of ENSO by both Niño3.4 and WHWP is 38.3%, that by Niño3.4 index only drops to 8.4%. It indicates that WHWP is able to contribute to additional 29.9% of ENSO variability with 95% confidence level by F-test. From this, it is known that WHWP in JAS(0) is independent from previous El Niño (D(-1)JF(0)), and able to predict El Niño in D(1)JF(2) with 17-month lag.


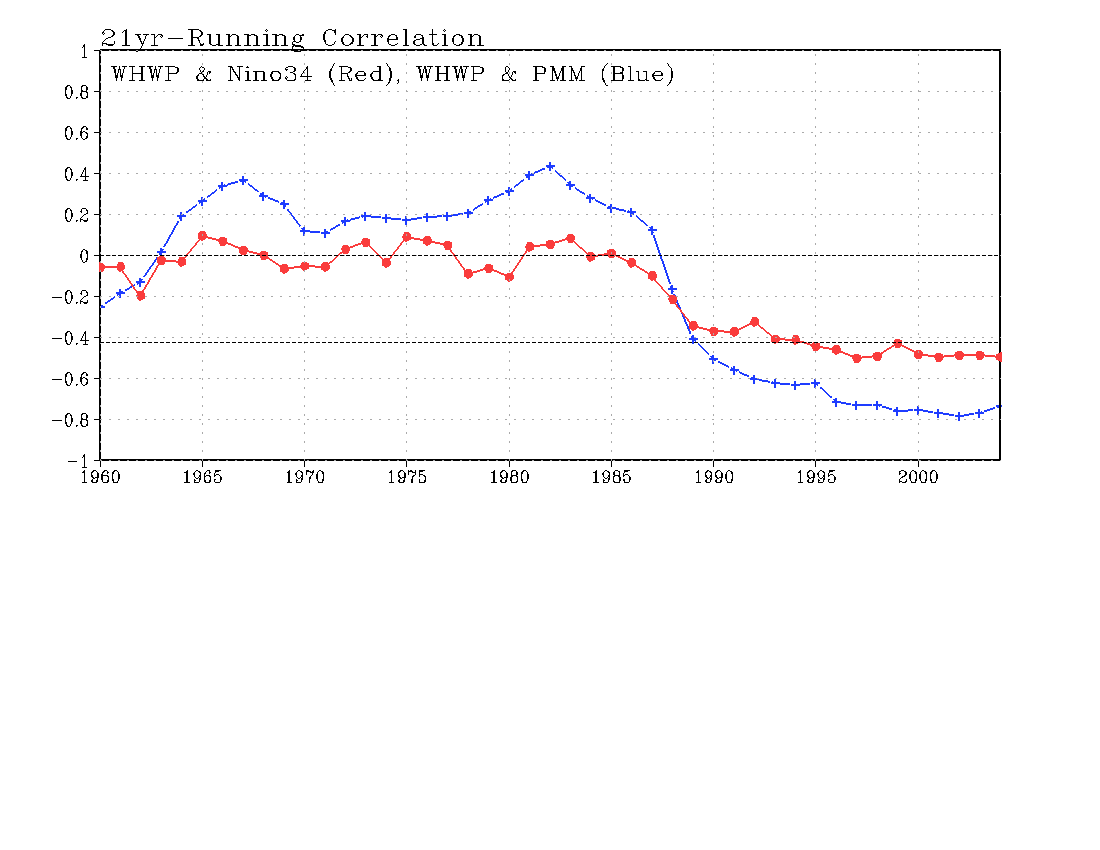


**Supplementary Fig 1** Relationship changes among WHWP, PMM, and Niño3.4. 21-year moving correlation coefficients of WHWP-Niño3.4 (Red), WHWP-PMM (Blue). Line of -0.43, marked by dotted line, indicates 95% confidence level (two-tailed t-test). Here, the PMM index is obtained by the following method in Chiang and Vimont (2007).


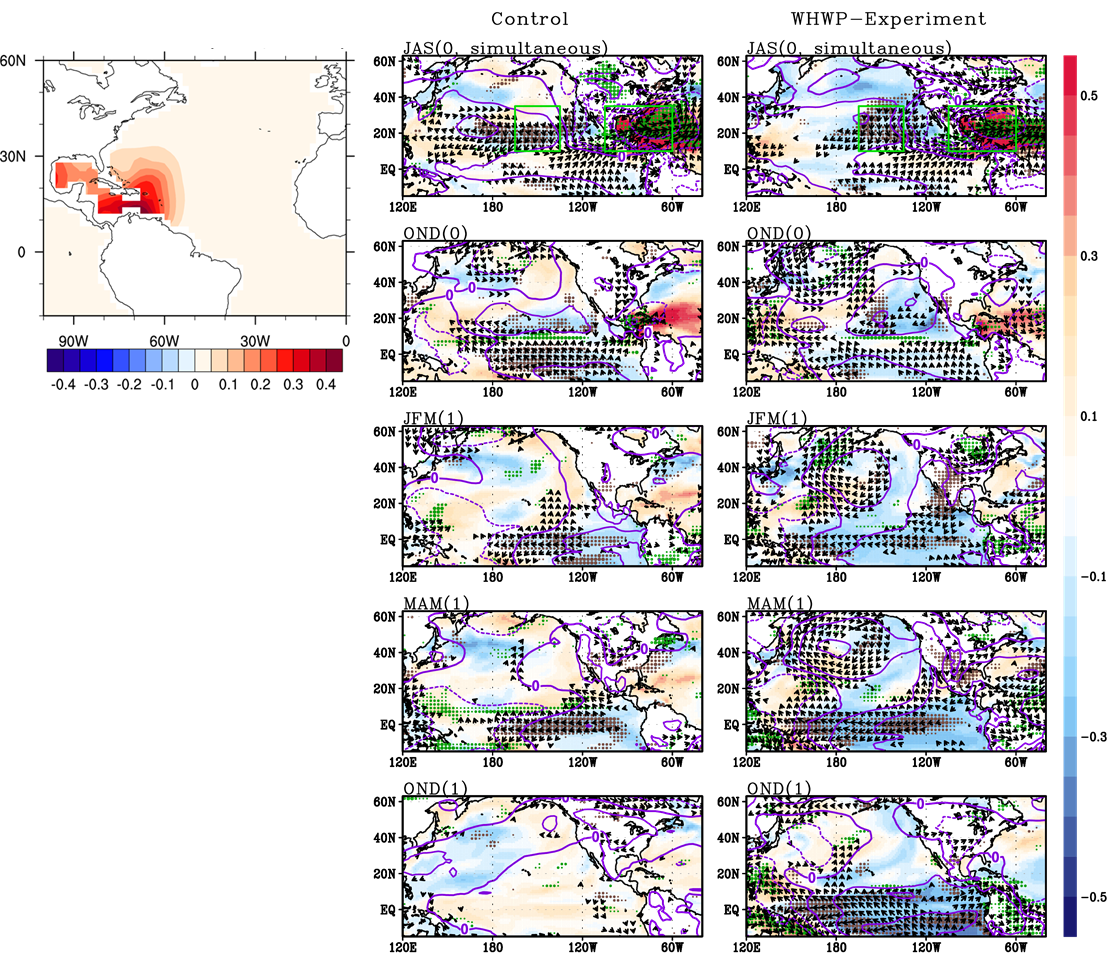


**Supplementary Fig 2** Climatological SST warming experiments in the WHWP with GFDL-CM2.1. Left figure shows climatological SST difference in the WHWP during JAS season between 1960-1985 and 1990-2015, which is applied to the WHWP-Experiment (right column) based on the Control-Experiment (middle column) by following method in Li and Hogan (1999). By doing so, higher SST climatology in the WHWP in JAS season can be obtained without the dampening of interannual variability. In the middle column, correlation maps of anomalous SST, SLP, precipitation, and winds against WHWP index (previous ENSO signal is removed) in the Control-Experiment (100yr) are shown during JAS(0) to OND(1), similar to the Fig. 2. In the figure, shading and vectors indicate SST and wind anomalies, and the brown and green dots show positive and negative precipitation anomalies, respectively. For precipitation and winds, above 90% confidence level is marked. Right column shows the same figures to the middle column, but with result from WHWP-experiment (100yr). From JAS(0) to MAM(1), anomalous northerly wind and SST cooling are more induced by the westward Rossby wave propagation associated with the WHWP in the WHWP-Experiment than control experiment. As a result, while there is no connection between WHWP-SST in JAS(0) and SST in the equatorial Pacific in OND(1) in the Control-Experiment, significantly enhanced connection between the two regions can be found in the WHWP-Experiment. These results are similar to those in the Fig. 2 based on the observation, which supports that the recent warming in the WHWP in JAS season plays an important role in the WHWP-El Niño connection.

#Li, T., & Hogan, T. F. The role of the annual-mean climate on seasonal and interannual variability of the tropical Pacific in a coupled GCM Journal of climate 12:780-792 (1999)
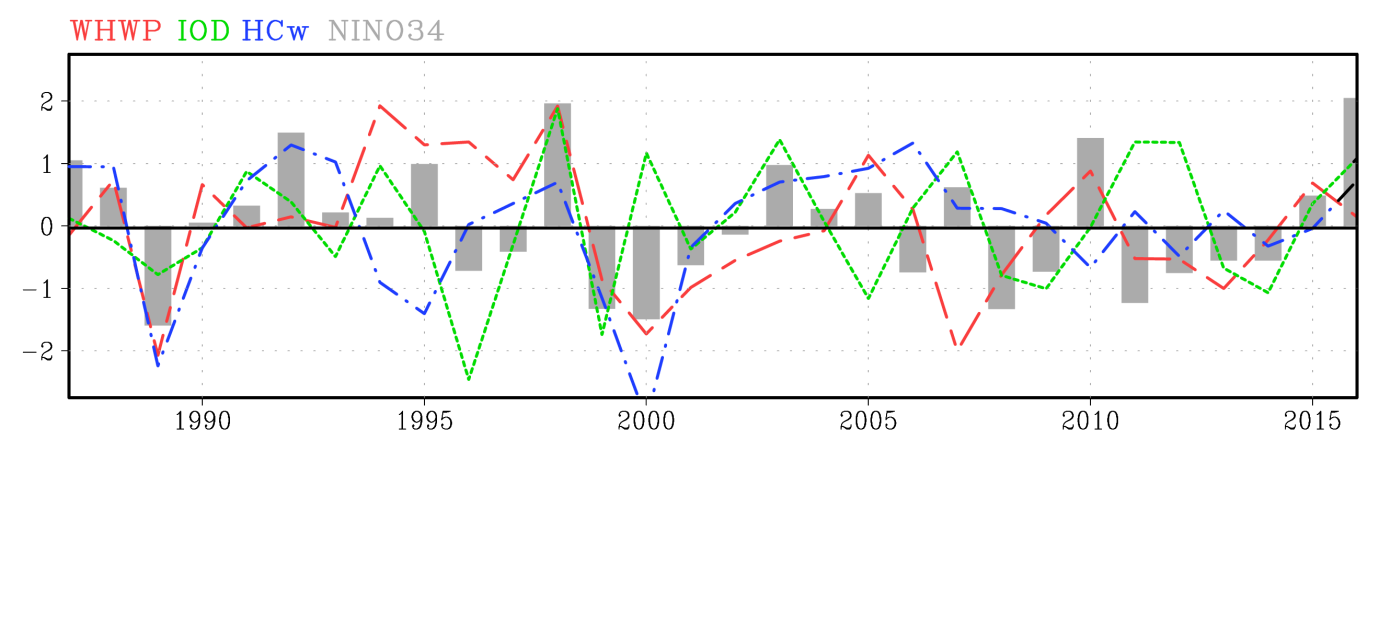


**Supplementary Fig 3** Various indices defined in this paper. Niño3.4(D1JF2), WHWP(JAS0), IOD(JAS0), and HC(JAS0) indices are indicated by the gray bar, red, green, and blue lines. In the figure, WHWP and IOD indices are multiplied by -1 for convenient comparison. The correlation coefficients among indices are like below table.

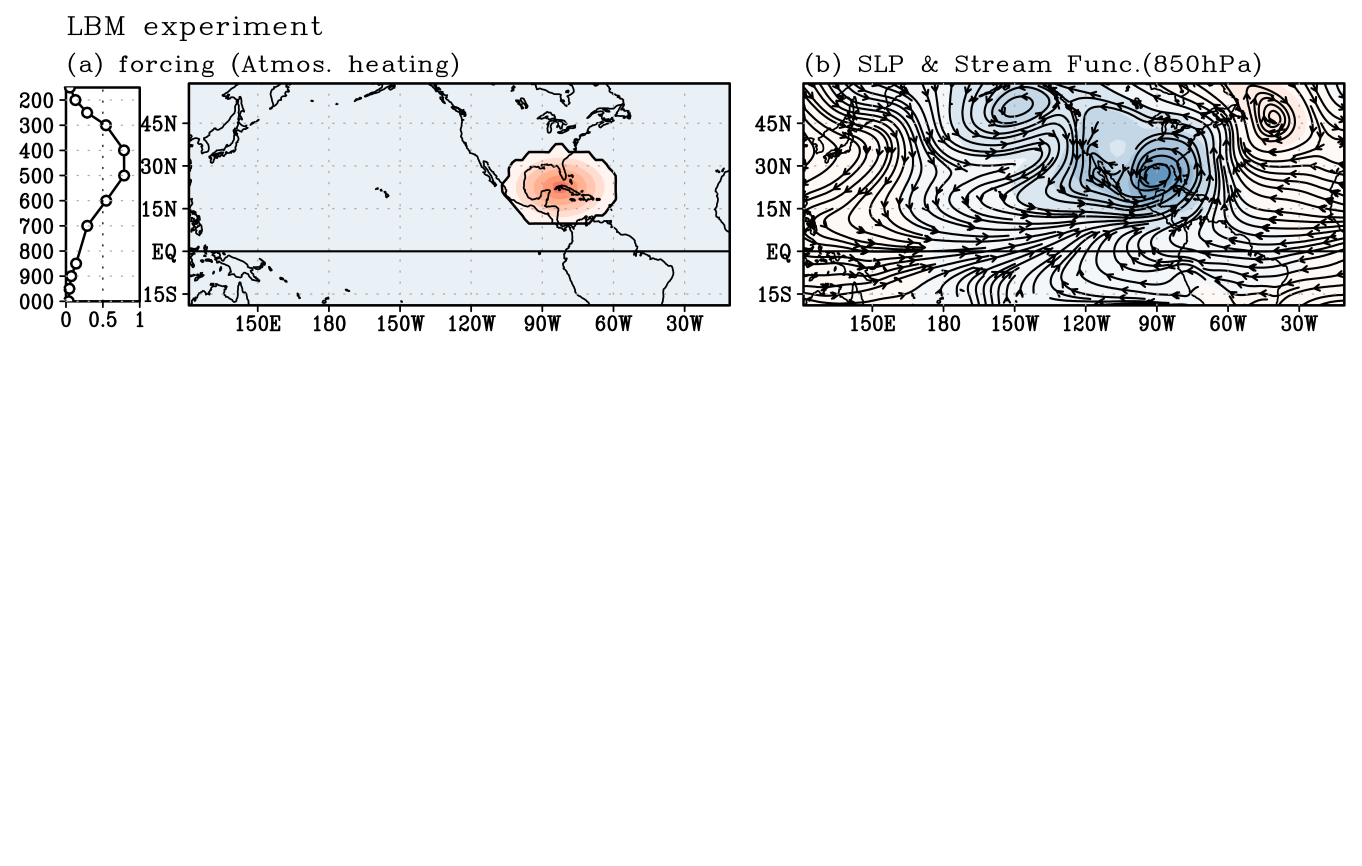


**Supplementary Fig 4** Linear baroclinic model experiment. (a) atmospheric heating forcing and (b) its steady responses of SLP (Shading) and stream function anomalies at 850 hPa from linear baroclinic model (LBM, Watanabe and Kimoto 2000) experiment under the atmospheric climatology in JAS season during 1985-2016. As a result of Rossby wave propagation from the heating forcing, anomalous northerly flows over the subtropical central North Pacific can be generated.

#Watanabe, M. and M. Kimoto, 2000: Atmosphere-ocean thermal coupling in the North Atlantic: A positive feedback. Quart.J.R.Met.Soc., 126, 3343-3369


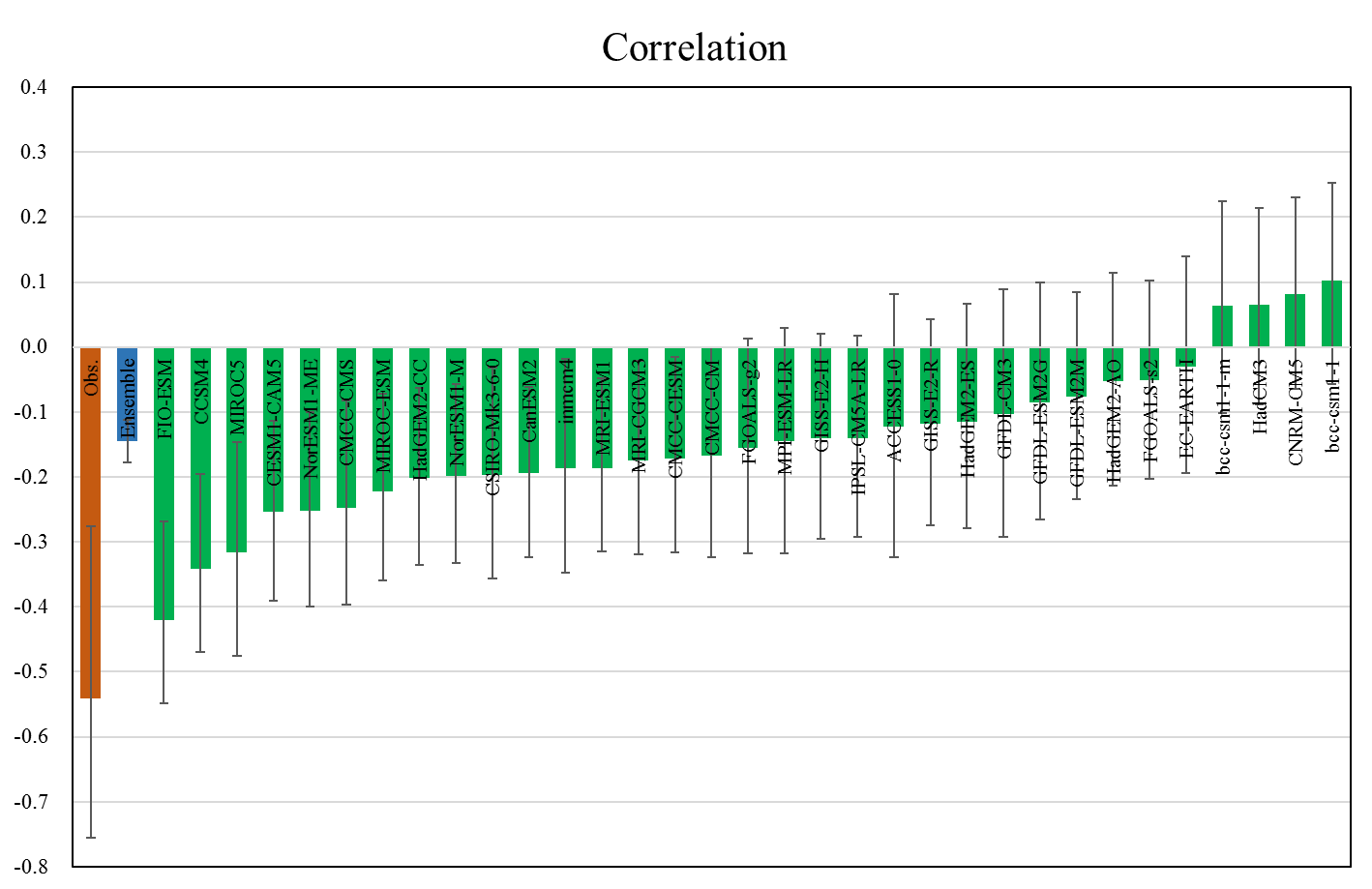


**Supplementary Fig 5** Lagged relationship between WHWP and Niño3.4 indices in CMIP5 models. Correlation coefficients between areal average of WHWP region in JAS0 and Niño3.4 indices in historical simulation (1970-2000) of each model from CMIP5. Left two columns show results of observation and model ensemble. Error bar indicates the 5 and 95% confident level using bootstrap method.


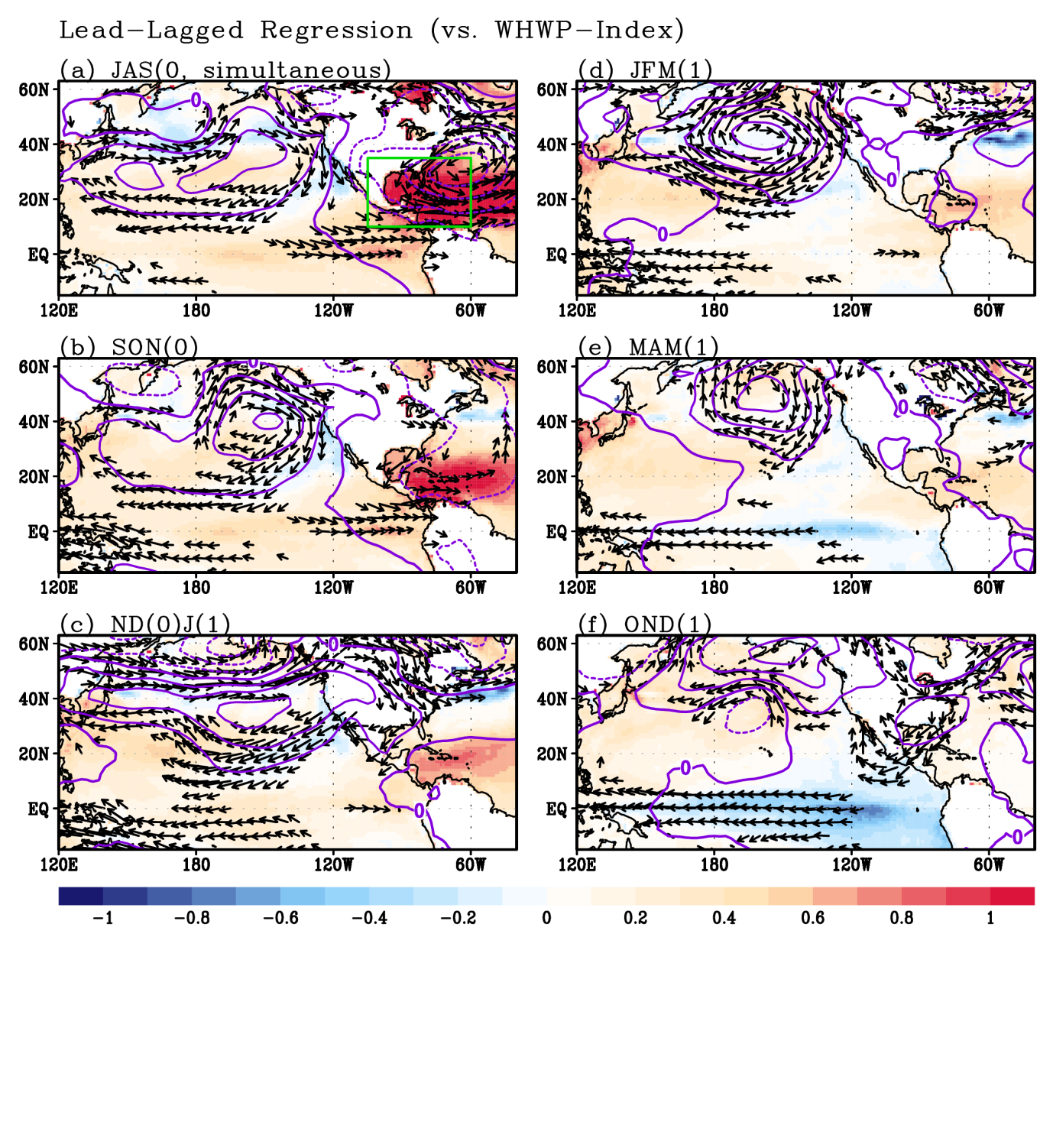
**Supplementary Fig 6** Lagged influence of WHWP on the Pacific (ensemble). Similar figure to the Fig. 2, but with ensemble results from historical runs (1970-2000). From (a) to (f) shows the time-sequential regression maps against to the WHWP index (previous El Niño signals are removed), where shading, contour, and vectors indicate anomalous SST, SLP, and wind, respectively.

**
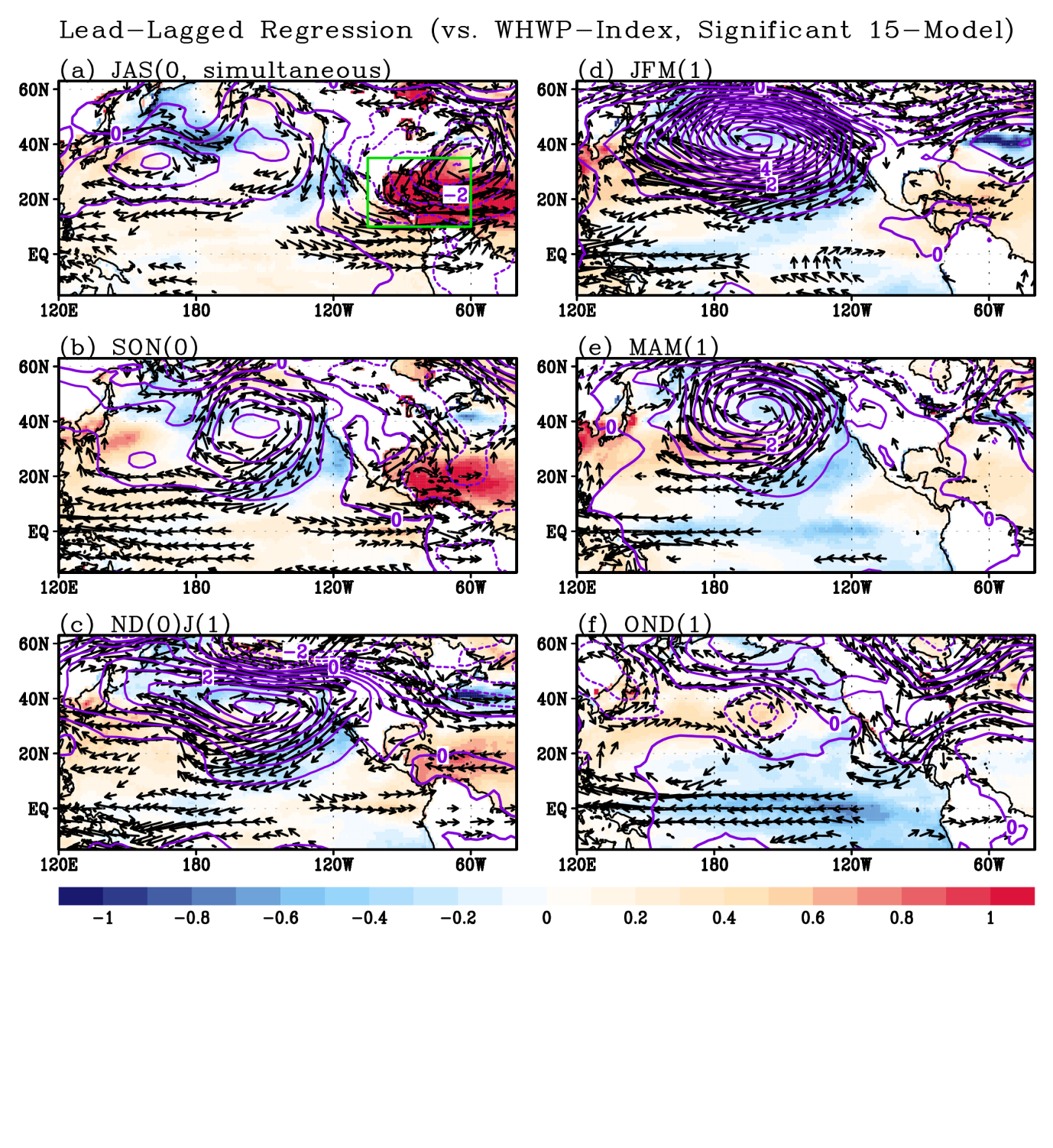
Supplementary Fig 7** Lagged influence of WHWP on the Pacific (significant 15-model).Similar figure to the Fig. 2, but with significant 15 model data based on Supplementary Fig. 4. From (a) to (f) shows the time-sequential regression maps against to the WHWP index (previous El Niño signals are removed), where shading, contour, and vectors indicate anomalous SST, SLP, and wind, respectively.

**
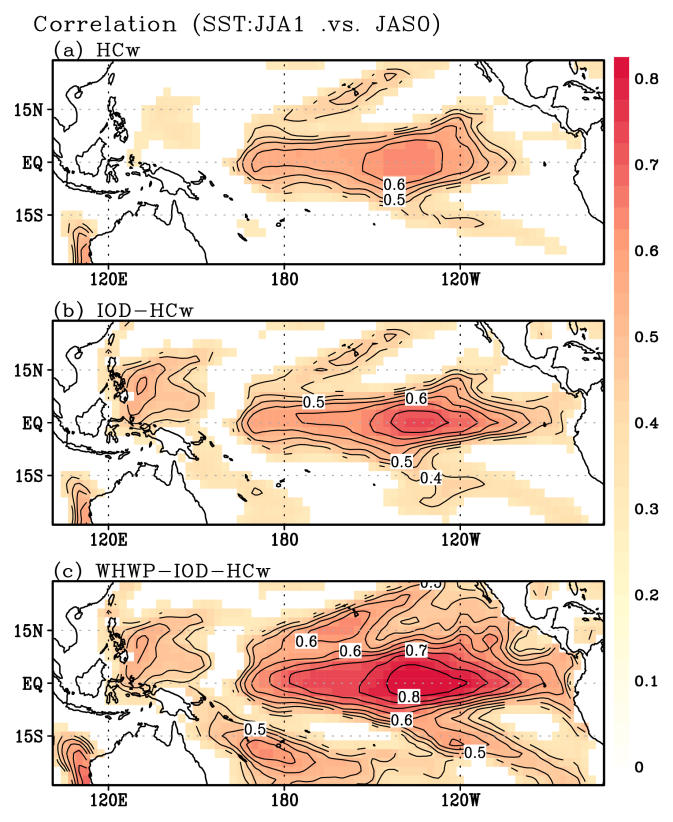
**
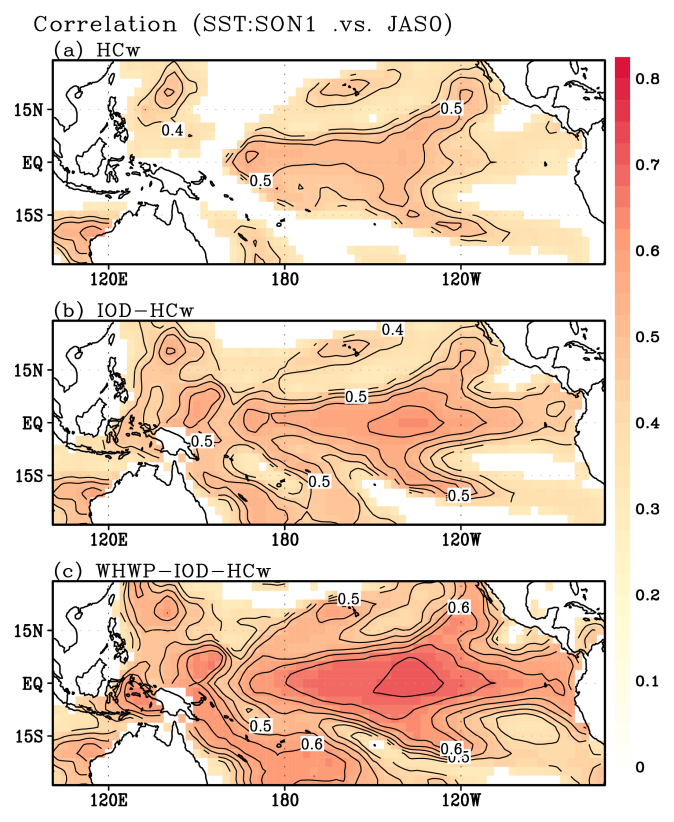


**Supplementary Fig 8** Grid-to-grid correlation maps between observed SST and Reproduced SSTA. The same figures to the Fig. 3, but with different seasons (left: JJA1, right: SON1).


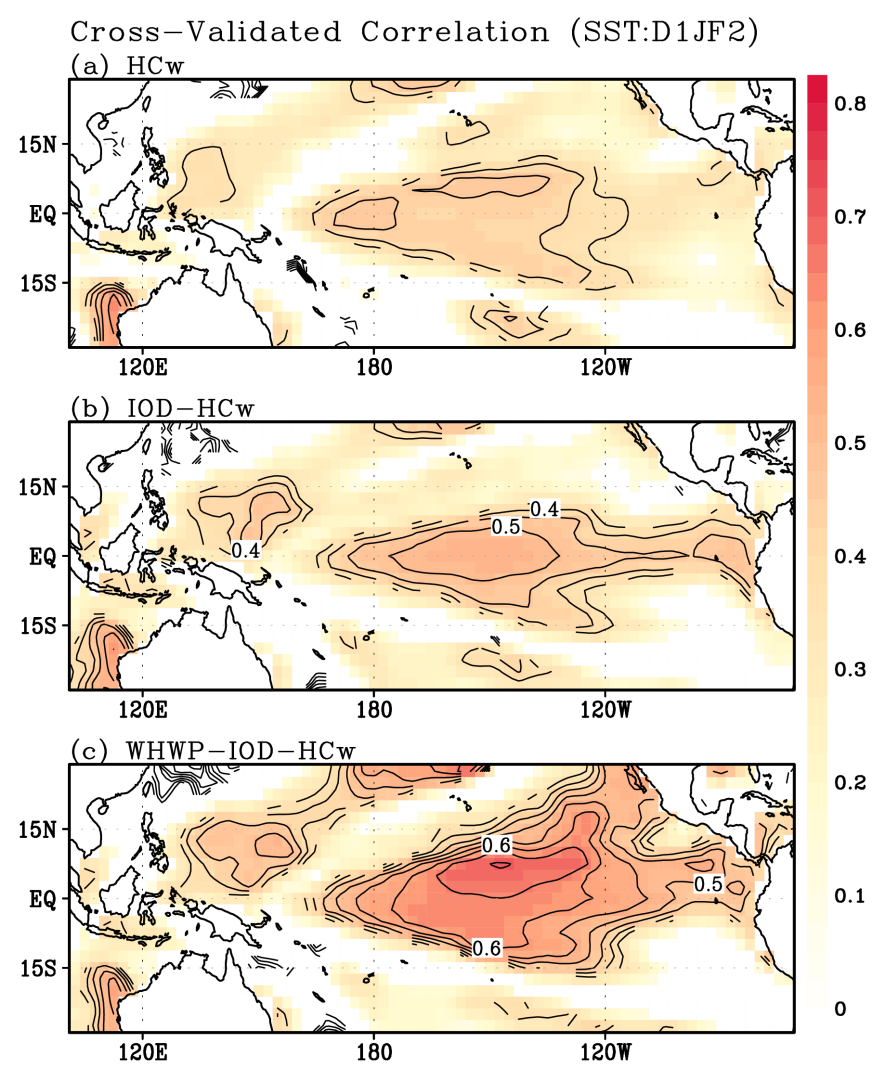


**Supplementary Fig 9** Grid-to-grid correlation maps between observed SST and Reproduced SSTA (cross validation). Same figure to the Fig. 3. Here, cross validation method is applied. (contour intervals are 0.05)

**
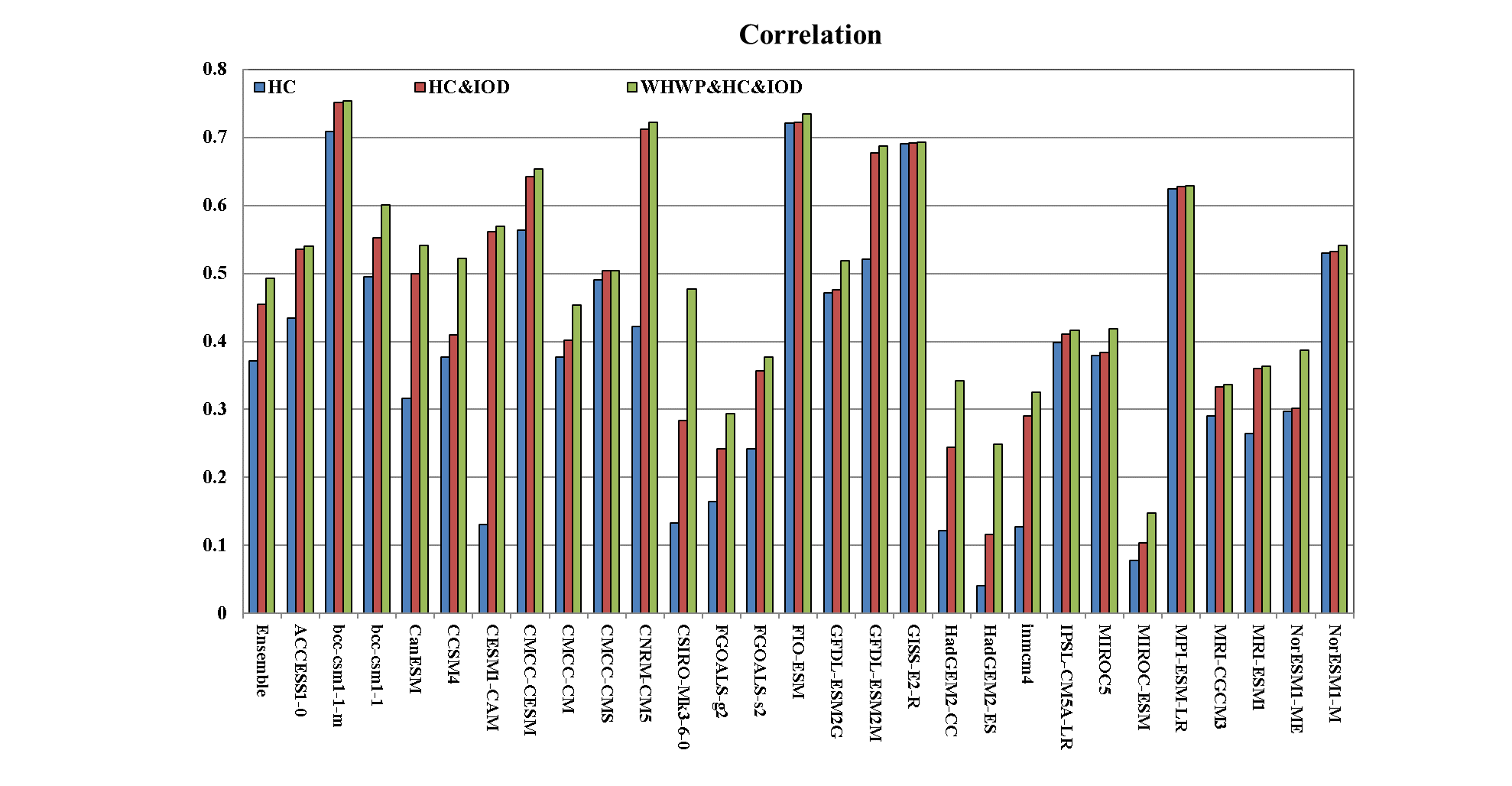
**

**Supplementary Fig 10** Correlation coefficients between original and modeled-reproduced Niño3.4 indices in CMIP5 models. The results of heat content only, heat content & IOD, and heat content & IOD & WHWP are indicated by blue, red, and green bar, respectively. Ensemble results are shown in the leftmost bar.
